# Causes Which Modify the Action of Anæsthetics—Dangers Incident to Anæsthetics

**Published:** 1861-03

**Authors:** 


### Selections.

Causes which Modify the Action of Anæsthetics—Dangers Incident to Anæsthesia.—*Lecture V, Tuesday, May* 29, 1860.—This lecture was commenced by the observation that age exercises a great influence on the action of an anæsthetic of any kind, either voluble or solid; young children are more speedily and entirely brought under the influence of narcotics than are adults or middle-aged persons. In old persons verging on their second childhood, narcotics, again, are speedy in their action, though not quite to the extent that they are in the young: this property extends to all anæsthetics. At the same time, it is worthy of notice that *volatile* narcotics are more safe to administer to children (on account of the quick respiration) than they are to adults, and no death has been known to occur from the administration of chloroform to children under five years of age. The solid narcotics, on the contrary, are very dangerous to administer to children.

The old are brought much more easily under the effects of chloroform than are middle-aged persons. There are, of course, exceptions to every rule. Females, as a rule, also are more speedily rendered insensible by chloroform than males; but here, again, there are exceptions. We may further set it down as a rule, that people of a strong physical formation, with a powerful, vigorous frame, are the most difficult to bring under the influence of an anæsthetic: Dr. Snow says, that the only case in which he ever had any difficulty in bringing a person under the influence of chloroform was an athletic. Much depends on the formation of the chest; for we find that a person with a small, narrow chest, and quick respiration, is very easily narcotised; with a broad-chested man the reverse obtains. Dr. Richardson once saw a young man rendered completely insensible in fifteen inspirations; and from the experience that he (Dr. R.) has had, he can generally tell by looking at a patient how much chloroform will be requisite to put the patient in an anæsthetic state. The action of anæsthetics may be modified by disease. Hysteria sometimes preduces a negative effect upon the action of chloroform. Dr. Snow always doubted this, never having had a case of the kind under his notice; but Dr. Richardson is in a position to prove it, having had a case, it appeared, in which hysterical sobbing during inhalation entirely prevented the insensibility. After trying for two hours ineffectually to bring the patient under the influence of the chloroform, he had to desist, as the heart was getting irregular. It is possible that in hysteria some alteration takes place in the composition of the blood, which prevents the absorption of the chloroform, thereby very much reducing the action of the anæsthetic.

With regard to the best means of administering chloroform in operations on the teeth, he (Dr. R.) is of opinion that a sitting position, with the head a little back, is the best. The inhalation in these operations is to be carried to the end of the third degree. Some persons operate during the first or second degrees; but as the greatest danger from chloroform arises in the first and second degrees, it is much the safest plan to push on to the third degree. Sometimes, indeed, in the second degree the mouth is firmly closed, owing to the contraction of muscle. In this case, no attempt to force the mouth open by means of a wedge, or in any other way, should be used, but the administration should be carried on until the third degree arrives; the jaw will then fall of itself. Dr. Richardson recommended a very good plan to the notice of the dentist particularly, as being simple, effective, and admirably adapted for cases of prolonged operations in the mouth, which the French have the merit of having introduced. It consists in having a tube fitted to the inhaler; after the patient is insensible, this tube is passed into one nostril of the patient, thereby causing him to inhale chloroform by one nostril, and air by the other.

A theory has been started by Dr. James Arnott (author of congelation), that in addition to the immediate dangers attending the administration of chloroform, there are secondary dangers, and even death three or four days after its administration, owing to shock, or the production of a low febrile state. Now, physiologically, this is not feasible; both Dr. Snow and Mr. Nunneley agree that chloroform has but one action—that of producing narcotism, and immediate death if carried beyond the proper limits. It can not possibly produce febrile symptoms; for after it is taken into the system, it undergoes no alteration, but remains as chloroform until it is passed out again; which transition takes place very rapidly, owing to the extreme volatility of the vapor. So far from chloroform causing shock to the system, Dr. Richardson argued that it prevented shock instead; and that now, with the aid of chloroform, many very serious operations, such as ovariotomy, amputations of the hip-joint and others, are frequently and successfully performed; whereas before the introduction of anæsthesia, such operations were hardly ever attempted, as the patient seldom recovered from the shock of the operation.

Most of the American dentists use ether in their operations, and there is no doubt but that it is less dangerous than chloroform—in fact, we have no very clear case of a death having arisen from the administration of ether. Mr. Nunn was supposed to have had a patient who died from its effects, but the proofs were faulty. Some persons use the argument, that ether has not been applied a sufficient number of times to thoroughly prove its effects; but if those persons would only take the trouble to make inquiries, they would find that it has been quite as extensively used as chloroform, though mostly in France and America. The greatest objection to ether lies in the fact that it is less ready, and not so determinate. Amylene would have every advantage of both chloroform and ether, if we could only get it pure. Dutch liquid has been used in about one hundred cases without a death occurring; but it has not been used a sufficient number of times to enable us thoroughly to judge of its qualities. The monochloruretted chloride of ethyle is thought by some to be better than chloroform; but that also has not been used sufficiently for us to arrive at an accurate conclusion.

The deaths that have occurred from chloroform are numerous. There is no denying that there have been at least one hundred and fifty cases of death, while many have occurred without any notice being taken of them. Dr. Richardson is aware of five unpublished deaths, and a friend of his of four more. Dr. Arnott says that if opium killed as many persons as chloroform, its use would be prohibited by law. The deaths occur either from the immediate effects of the chloroform, or from the too long administration of it; generally from the first of these two causes, the second seldom occurring, unless through carelessness. In one case, a lady administered it to herself during her confinement, and died from excess of inhalation.

There has been much discussion and theory as to the immediate cause of death from chloroform. Some think that it is through spasm of the glottis; some, by asphyxia; others, that it is caused by the tongue dropping back upon the glottis, and the like. The fact is, there has been no precision in the examinations of patients dying from chloroform; for every one about the patient is alarmed, the galvanic apparatus or artificial respiration is applied, and every one is so anxiously endeavoring to preserve life, and no one is taking minute observations of the precise symptoms of the dying patient—therefore it is impossible to tell with any certainty from the human subject the actual cause of death. All the facts we have, or nearly all, are derived from experiments upon animals. Dr. Richardson has performed a number of experiments with various animals, and the conclusion he has come to with regard to the reason of death is, that it takes place through paralysis of the heart, the view first advanced by Dr. Sibson. He (Dr. Richardson) injected some chloroform into the aorta, and so into the coronary arteries of an animal; immediate paralysis of the heart was the result. In one experiment, he removed the heart from the body, and placed it, still beating, on the table. On bringing the vapor of chloroform to bear upon it, the contractions stopped immediately. There is no doubt, indeed, but that chloroform has a direct action on the heart, being brought to it by the blood. At its first introduction into the organ (in the first degree of anæsthesia), the vapor sometimes causes over-excitement of the organ, and death. One fact that goes a great way to prove this theory is, that out of fifty cases of deaths from chloroform recorded by Dr. Snow in his work, all with but three exceptions died from cardiac syncope, or other form of heart disease, and all except five died within five minutes. The deaths were nearly all in cases of small operations, eighty per cent. being in teeth extractions; many of the deaths occurred before the operation was attempted.

Some object to the administering of chloroform in tubercular diseases of the lung, such as tubercle; but Dr. Snow has administered it with success to a great number of patients afflicted with tubercular disease. Dr. Richardson has done the same, and considers that, as a rule, no class of persons take it better; at the same time, he does not dispute that there might be cases where it would not be advisable to administer it. In all cases, the organ we have most to consider is the heart. Dr. Richardson here gave a condensed account of the anatomy and physiology of that organ, and the circulation of the blood through it. He then spoke of the pathology of the heart, explaining how there are two kinds of diseases, viz., valvular disease and structural disease. In the first, the valves do not perform their functions properly; the second includes hypertrophy of the heart; thinness or weakness in any portion of the muscular wall; and softening of the heart, as from fatty degeneration. All these diseases may be discovered by auscultation; and it is worthy of notice that it is not the valvular, but the structural disease that is the most dangerous.

In conclusion, Dr. Richardson called attention to a few of the leading signs by which we might be able to judge as to whether a patient was in a fit state to receive chloroform. They were as follows:—If we find that the patient is generally feeble, with a pulse whose beat is feeble, depressed, and irregular—or if we find a very plethoric condition of body, we should be chary in attempting to administer the narcotic without first examining the patient by auscultation. If the result of that proves unmistakably that the patient is suffering from disease of the walls of the heart, we must object to the administration of any anæsthetic.—*Dr. Richardson's Lectures, reported for the Dental Review.*

